# Trajectories of leisure reading for children with literacy difficulties and the role of literacy support: Findings from the UK millennium cohort study

**DOI:** 10.1111/bjep.70113

**Published:** 2026-08-05

**Authors:** Miriam McBreen, Francesca Bentivegna, Emma Sumner, Efstathios Papachristou

**Affiliations:** ^1^ University College London London UK; ^2^ University of Groningen Groningen the Netherlands; ^3^ Liverpool John Moores University Liverpool UK

**Keywords:** leisure reading, literacy, literacy intervention, millennium cohort study, reading, reading motivation

## Abstract

**Background:**

Evidence suggests leisure reading may be especially important for children with reading difficulties. However, little is known about how their leisure reading evolves over time or how school‐based practices, including targeted literacy support, impact this.

**Aims:**

We aimed to examine how literacy difficulties are associated with leisure reading concurrently and over time for upper primary children with literacy difficulties, and whether receiving literacy support moderated this relationship.

**Sample:**

Our sample included 7291 participants, using longitudinal data from the Millennium Cohort Study (MCS), a UK nationally representative longitudinal birth cohort study.

**Methods:**

Chi‐squared tests were conducted to explore associations among main variables (literacy difficulties, literacy support and leisure reading at 11, 14 and 17). Latent growth curve models were used to analyse associations between literacy difficulties and the trajectory of leisure reading, including the moderating effect of receiving literacy support.

**Results:**

Literacy difficulties were significantly associated with less frequent leisure reading at all ages, but not with the trajectory of leisure reading over time. Receiving literacy support was not associated with leisure reading at any time point and did not moderate the association between literacy difficulties and leisure reading.

**Conclusions:**

The findings presented here suggest that children with literacy difficulties show lower leisure reading than those without literacy difficulties throughout upper primary and secondary years, and that their leisure reading declines whether or not they have received targeted literacy support. This indicates a shift is needed to effectively support these children.

## INTRODUCTION

Reading difficulties are among the most common learning difficulties, with prevalence estimated between 4% and 10% (Deighton et al., 2020). Their effects are wide‐ranging, including on academic achievement (Boyes et al., 2016; Graham et al., 2021), well‐being, school attendance (O'Reilly & McNamara, 2007) and employability (Bynner & Parsons, 2006). While extensive research has examined how best to support children with reading difficulties (Al Otaiba & Fuchs, 2002; Boucher et al., 2024; Hall et al., 2023), the focus has tended to be on how to improve the skills needed for successful reading (Miyamoto et al., 2020). However, there is now growing recognition that to support best outcomes for these learners, both academically and socio‐emotionally, instruction should also support reading motivation and leisure reading (Cho et al., 2023; McGeown & Wilkinson, 2021).

The need to foster reading motivation is reflected in national curriculums across the world (e.g., in New Zealand; Milne et al., 2023 and Australia; Vanden Dool & Simpson, 2021). In the United Kingdom, where this research was conducted, curricula of all four nations emphasize that children should have opportunities to develop a habit of reading for pleasure (CCEA, 2019; DfE, 2023; Scottish Government, n.d.; Welsh Government, 2016). Yet, the number of children who read for pleasure is declining, with UK children's reading attitudes and leisure reading at their lowest point since records began in 2005 (Clark et al., 2024). The development of concrete recommendations to address this issue has been hindered by a lack of research into how school practices affect leisure reading. In particular, there is a need for more research that examines how leisure reading develops over time for children with reading difficulties, and that examines how school‐based targeted literacy support contributes to this relationship.

### Reading ‘skill’ and ‘will’

Reading achievement and motivation are closely linked throughout school years (e.g., Toste et al., 2020), with higher motivation linked to improved reading comprehension (Guthrie & Wigfield, 2000; Taboada et al., 2009). Intrinsic motivation to read (i.e., engaging in reading for personal enjoyment or perceived significance; Covington & Müeller, 2001) may be especially important. Indeed, high levels of intrinsic motivation are linked to greater progress in reading compared to high levels of extrinsic motivation (i.e., reading to meet internal feelings of pressure or comply with demands) (De Naeghel et al., 2012). In part, this may be due to the impact on reading behaviours; children with high intrinsic motivation tend to use more strategies during reading and read more during their leisure time (De Naeghel et al., 2012; Miyamoto et al., 2020). Leisure reading, or reading for pleasure, refers to reading we engage in of our own free will, driven by interest or the satisfaction of reading (Clark & Rumbold, 2006). Frequent leisure reading has been linked to progress in reading (Sullivan & Brown, 2015; Torppa et al., 2020), but also to a wider range of benefits, including in maths (Sullivan & Brown, 2015), language (Nation et al., 2022), empathy (Bal & Veltkamp, 2013), well‐being (McGeown & Wilkinson, 2021) and adoption of healthy behaviours (Mak & Fancourt, 2020).

Understanding how to foster intrinsic motivation may be especially important for children with reading difficulties, who are more likely to experience low motivation and less likely to read for pleasure (Nelson & Manset‐Williamson, 2006). Research indicates that in the early stages of learning to read, reading ability determines how likely children are to read for pleasure, while in later years, reading for pleasure both results from and contributes to reading skills (Torppa et al., 2020; van Bergen et al., 2023). For these learners, reduced leisure reading may further exacerbate difficulties through limiting opportunities to develop reading skills (Morgan & Fuchs, 2007). Indeed, there is evidence that as children reach upper‐primary age, the impact of intrinsic motivation is most important for those with reading difficulties. For instance, in a sample of 9‐ to 11‐year‐olds, Logan et al. (2011) found that intrinsic motivation explained significant variance in progress in comprehension skills for children with low reading ability, but not for those with high reading ability. However, less is known about how the relationship between reading difficulties and leisure reading evolves over time.

### Reading supports and motivation

One area of school practice that merits further investigation is the impact of targeted literacy support for upper primary‐aged children with literacy difficulties. Evidence suggests that access to early and intensive reading intervention is essential for children with reading difficulties, as regular classroom instruction may be insufficient (e.g., Vaughn & Wanzek, 2014). This approach is reflected in all UK curriculums, which recommend a combination of high‐quality teaching and, where necessary, targeted interventions to support learners experiencing literacy difficulties (DfE, 2015; DfE NI, 2011; Scottish Government, n.d.; Welsh Government, 2016). However, regardless of early intervention, some children experience literacy difficulties that continue into upper primary years and need ongoing additional support (Austin et al., 2017). Importantly, how targeted literacy support affects reading motivation for these children remains unclear (Miyamoto et al., 2020).

Over the past 20 years, the dominant approach to teaching reading in the United Kingdom has been through Systematic Synthetic Phonics (SSP) (Solity, 2022). Whole class teaching practices in primary school typically take a phonics‐based approach to teaching reading (DfE, 2023), while interventions that aim to support struggling readers will build on early whole class approaches (e.g., phonology‐based intervention) but in smaller groups or on a one‐to‐one basis with greater intensity (DfE, 2023; Fuchs & Fuchs, 2006). There is considerable evidence that reading interventions that are explicit, systematic and focused on code (e.g., phonics) and meaning contribute to improving foundational reading skills for children with reading difficulties (e.g., Al Otaiba & Fuchs, 2002). However, how these interventions impact motivation has been the subject of considerable debate, with critics arguing that a focus on SSP negatively impacts motivation by limiting learners' opportunities to engage with real texts and find pleasure in reading (Solity, 2022; Wyse & Bradbury, 2022). On the other hand, proponents argue that by enabling children to experience success in reading, SSP approaches build skills, confidence and, as a result, foster motivation (DfE, 2023).

Potentially, improving skills will also improve motivation, feeding into a virtuous cycle between skills and motivation. Some evidence supports this idea. For instance, Bates et al. (2016) reported that motivation partly mediated gains in achievement following a Reading Recovery intervention, and Chen and Savage (2014) found that a reading intervention focused on rapidly building foundational skills, through teaching high‐frequency phoneme‐to‐grapheme correspondences, positively impacted both achievement and motivation. Findings showing that reading skills fuel reading enjoyment in the early years (van Bergen et al., 2023) also suggest that interventions targeting skills may contribute to more frequent leisure reading. Conversely, other research has found that interventions focused exclusively on skills have no effect on motivation (Morgan et al., 2008; Quirk & Schwanenflugel, 2004) and, in some cases, can lead children to adopt more negative attitudes towards reading (Wanzek et al., 2006). Given these contradictory findings, more research is needed to ascertain how the additional support children receive in schools affects their reading motivation and leisure reading. In particular, research that looks at practices happening in schools, rather than within tightly controlled experimental studies, is needed to better understand practice in real‐world contexts and inform recommendations (Troyer, 2022).

### The present study

In the present study, we aimed to examine how literacy difficulties in upper primary school (at age 11) relate to concurrent and later leisure reading, and whether receiving literacy support moderated this relationship. While most research on literacy motivation has focused on reading specifically, we took a broad approach that included all children with literacy difficulties (i.e., in reading and/or writing), as children with reading difficulties have been shown to experience broader challenges with literacy (Graham et al., 2021).

Specifically, we aimed to address the following research questions:

1. How are literacy difficulties at age 11 and leisure reading associated, both concurrently and over time?
2. Does receiving literacy support moderate the relationship between literacy difficulties and leisure reading, both concurrently and over time?

## METHODS

### Sample

We used longitudinal data from the Millennium Cohort Study (MCS), a UK nationally representative birth cohort study of 19,243 families whose data was first collected between 2000 and 2002. We included information from the first 7 sweeps, which cover school age, starting with 18,522 cohort members (aged 9 months) in Sweep 1, and ending with 10,625 members (aged 17 years) in Sweep 7. Data for the main variables is available from Sweep 5 (age 11). Participants were included if they had data on at least one measurement of leisure reading (age 11, 14 or 17). A total of 7291 participants (50% female, 81.9% White, 73.7% with a family income above the 60% median) were included in the sample. Of these participants, 83.7% came from England, 15.6% from Wales, .5% from Scotland and .2% from Northern Ireland. Relevant covariates from Sweep 1 to Sweep 4 were also included as appropriate (detailed in the measures below). For each sweep, ethical approval was obtained from the NHS Multi‐Centre Ethics Committee.

### Measures

#### Primary measures

##### Literacy difficulties

Assessed at age 11 using a single item on a questionnaire administered to teachers, who were asked: ‘*In so far as your professional experience will allow, please rate this child in relation to all children of this age (i.e., not just their present class or, even, school)?*’ for English (or Welsh) reading and writing, rated on a 5‐point Likert scale ranging from ‘Well above average’ to ‘Well below average’. The variable was treated as binary with ‘below average’ and ‘well below average’ categorized as ‘literacy difficulties’ and all other answers categorized as ‘no literacy difficulties’.

##### Literacy support

Assessed at age 11 using a single item on a questionnaire administered to teachers, who were asked ‘*Does the child get any support in English, Reading, Writing, Literacy, Phonics?’*, to which they could answer yes or no.

##### Leisure Reading

Measured at ages 11, 14 and 17 with a single question to the cohort members asking how often they read for pleasure. The questions at each time point were similar (e.g., ‘*How often do you read for enjoyment, not for school?*’; *‘How often do you do the following activities? [Read for enjoyment?]’*), and the answers were recoded so that the frequency of leisure reading was rated on a 5‐point Likert scale going from ‘Never’ to ‘Most days’.

#### Potential confounds

Finally, the following variables were included in the analyses as potential confounds, in line with existing literature suggesting their associations with the main variables:

##### Socio‐demographic variables

Including sex (male vs. female), ethnicity (White vs. Other than White), household income (below vs. above 60% median poverty indicator), maternal education (higher education vs. lower education).

##### Earlier Reading/writing contributing variables

Including home learning environment (age 3) (Claes et al., 2024), number of books in the home (age 7) (Cole et al., 2022), previous reading enjoyment (age 7) (Torppa et al., 2020), reading accuracy (age 7) (van Bergen et al., 2023), cognitive ability (age 5) (Hulslander et al., 2010; Sun et al., 2024) and emotional and behavioural problems (age 7) (Sun et al., 2024). The home learning environment was measured using the ‘home learning environment index’, created based on frequency of activities linked to literacy and numeracy in the home environment, such as reading to the child, going to the library, painting, drawing, teaching letters and numbers, and teaching songs, poems and rhymes (de la Rochebrochard, 2012). Cognitive ability was assessed using three subscales (Pattern similarities, Naming vocabulary, Pattern construction) from the British Ability Scale‐2nd Edition (BAS‐II; Elliott et al., 2004) and was then standardized following principal component analysis. Reading accuracy was assessed through the BAS‐II. Lastly, emotional and behavioural problems were assessed using the total difficulties score of the Strengths and Difficulties Questionnaire (Goodman, 1997).

### Statistical analyses

We used Stata v.18.0 to clean the data and run descriptive analyses, including chi‐square tests and sample bias analyses, while Mplus v.8 was employed for the latent growth curve modelling. A Full Information Maximum Likelihood (FIML) approach with robust standard errors was used to deal with missing data and boost the sample size, and the relevant weights, stratification and cluster points were added to manage the disproportionate design of MCS.

Preliminary analyses included exploration of sociodemographic characteristics of the sample, including sample bias analysis. Chi‐squared tests were employed to explore the significance of the associations among the main variables. Next, latent growth curve models were fitted to examine trajectories of leisure reading across ages 11, 14 and 17. Latent growth curve models were deemed more appropriate than other approaches (e.g., growth mixture models) due to the small number of children in the analytic sample who had received literacy support. We first estimated a trajectory‐only model to derive the latent intercept and slope of leisure reading. The intercept represented the initial level of leisure reading at age 11, and the slope represented linear change in leisure reading from age 11 to age 17.

We then fitted conditional latent growth curve models to test whether literacy difficulties at age 11 were associated with the intercept and slope of leisure reading. Next, literacy support at age 11 was added to the model to examine whether receiving support moderated the association between literacy difficulties and the intercept and slope of leisure reading. Models were first fitted without adjustment and then with adjustment for key covariates in the minimally adjusted model. The key covariates were sex, ethnicity, family income, home learning environment, previous reading enjoyment and reading accuracy. As FIML allows participants with incomplete data to contribute to the estimation, the inclusion of additional covariates did not reduce the sample size, and the analytic sample remained constant across the unadjusted and minimally adjusted models.

As sensitivity analyses, we re‐ran the same models using a complete case sample. Model fit indices were examined for each model. Specifically, RMSEA ≤.08, CFI/TLI ≥.90 and SRMR ≤.08 were considered to indicate adequate to good fit. Fit indices can be found in Table S1 for latent growth models and Table S2 for the complete case sensitivity analysis. An overview of the model including the interaction with literacy support is presented in Figure 1.

**FIGURE 1:**
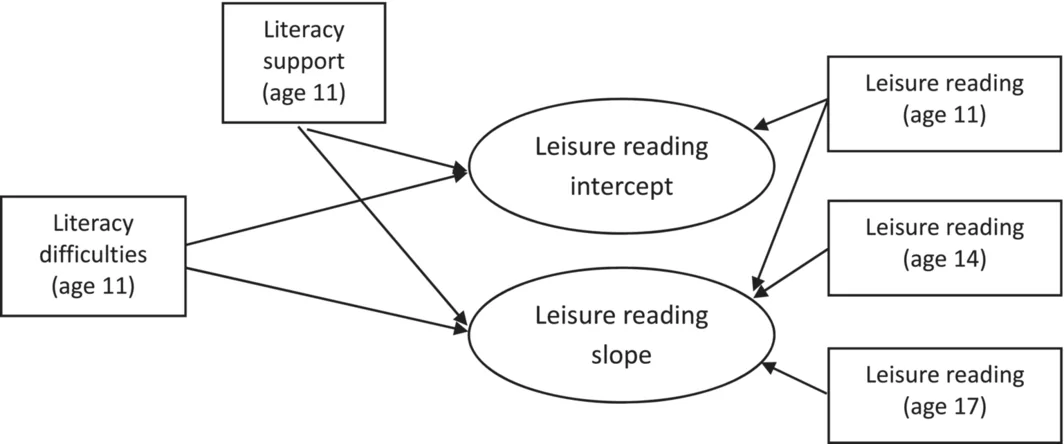
Overview of the model including the interaction with literacy support. Literacy support (Age 11). Model with literacy difficulties at Age 11 as the exposure, literacy support as the moderator (or interaction term), and the trajectories (intercept and slope) of leisure reading as the outcome.

## RESULTS

### Descriptive and sample bias analyses

The analytic sample (i.e., those included in the analysis) comprised 7291 participants (see Table 1 for descriptive statistics, including level of missingness for all variables). Missing data were present for most variables, with the highest proportion for leisure reading at age 17 (47.7%). Missing data were addressed through FIML as detailed above. The proportion of participants who presented with literacy difficulties at age 11 was 20.23% (*n* = 1463). Among those with literacy difficulties, 2.3% (*n* = 33) received literacy support at 11. Among those without literacy difficulties, .6% (*n* = 32) received literacy support at 11 (Table S3).

**TABLE 1:** Socio‐demographic characteristics of the analytic sample and covariates.

Exposure and outcomes	Analytic sample	
	Sample *N* (%)	Missingness *N* (%)—Out of 7291
**Literacy difficulties (Age 11)**		58 (.8)
No literacy difficulties	5770 (79.8)	
Literacy difficulties	1463 (20.2)	
**Literacy support (Age 11)**		252 (3.5)
No reading support	6974 (99.1)	
Reading support	65 (.9)	
**Leisure reading (Age 11)**		81 (1.1)
Never of almost never	483 (6.7)	
Less often than once a month	518 (7.2)	
At least once a month	719 (10.0)	
At least once a week	2109 (29.2)	
Most days	3381 (46.9)	
**Leisure reading (Age 14)**		1167 (16.0)
Never of almost never	1122 (18.3)	
Less often than once a month	1486 (24.3)	
At least once a month	1073 (17.5)	
At least once a week	1092 (17.8)	
Most days	1351 (22.1)	
**Leisure reading (Age 17)**		3475 (47.7)
Never of almost never	1078 (28.3)	
Less often than once a month	1203 (31.5)	
At least once a month	553 (14.5)	
At least once a week	472 (12.3)	
Most days	510 (13.3)	
**Covariates (binary/categorical)**		
**Child's sex**		0 (0)
Male	3647 (50.0)	
Female	3644 (50.0)	
**Child's ethnicity**		0 (0)
White	5969 (81.9)	
Other than white	1322 (18.1)	
**Family income** ^a^		581 (8.0)
Above 60% median	4944 (73.7)	
Below 60% median	1766 (26.3)	
**Maternal education** ^a^		575 (7.9)
No higher education	4117 (61.3)	
Higher education	2599 (38.7)	
**Number of books at home** ^b^		90 (1.2)
0–10	935 (13.0)	
11–25	970 (13.5)	
26–100	2358 (32.8)	
101–200	1472 (20.4)	
201–500	1023 (14.2)	
More than 500	443 (6.1)	
**Reading enjoyment** ^a^		1040 (14.3)
Don't like it	682 (10.9)	
Like it a bit	1943 (31.1)	
Like it a lot	3626 (58.0)	
**Covariates (continuous)**	**M (SD)**	***N* (%)—out of 7291**
Home learning environment^c^	26.92 (6.90)	1147 (15.7)
Child's cognitive ability^d^, ^e^	100.06 (14.93)	533 (7.3)
Reading accuracy^a^, ^e^	100.02 (15.00)	739 (10.1)
Mental health difficulties total score^a^	6.98 (4.98)	676 (9.3)

Abbreviations: %, percentages with number of non‐missing values as the denominator; *M*, mean; *N*, total number of cohort members; SD, standard deviation.

^a^ Measured at age 7.

^b^ Measured at age 11.

^c^ Measured at age 3.

^d^ Measured at age 5.

^e^ Standardized.

Some statistically significant differences were present between the analytic sample and the non‐analytic sample (i.e., the full cohort of children included in the MCS; *N* = 11,952), as displayed in the sample bias analysis in Table S4. Compared to the analytic sample, the non‐analytic sample included a higher proportion of participants with literacy difficulties at age 11 (*x* ^2^ = 27.6, *p* < .001), but a lower proportion of participants who frequently read for leisure (*x* ^2^ = 31.7, *p* < .001). The difference in literacy support was not significant. As for the covariates, in the non‐analytic sample there were less female cohort members (*x* ^2^ = 9.3, *p* < .1), more families with lower income (*x* ^2^ = 87.3, *p* < .001), less mothers with a higher education (*x* ^2^ = 53.45, *p* < .001), a lower number of books at home (*x* ^2^ = 66.64, *p* < .001), a lower mean home learning environment index (*x* ^2^ = 37.6, *p* < .001), lower cognitive ability (*x* ^2^ = 92.96, *p* < .001) and reading accuracy (*x* ^2^ = 171.41, *p* < .001), and higher emotional and behavioural problems (*x* ^2^ = 19.92, *p* < .01).

### Association of literacy difficulties and leisure reading

We first sought to examine whether literacy difficulties were associated with leisure reading, both concurrently and over time. In our overall analytic sample, which included both children with and without literacy difficulties, almost half (46.9%) read for pleasure most days at age 11, while this percentage gradually dropped at ages 14 (22.1%) and 17 (13.4%). At 17, most children (59.8%) reported they read for pleasure ‘less often than once a month’ or ‘never or almost never’, compared to 42.6% at age 14 and 13.9% at age 11.

Chi‐squared tests of association showed that literacy difficulties at age 11 were significantly associated with leisure reading at all time points (ages 11, 14 and 17), with leisure reading lower at all time points for pupils with literacy difficulties compared to those without literacy difficulties (Table S3).

For results from the latent growth curve models, the intercept and slope of leisure reading at ages 11, 14 and 17 were derived, with the intercept being 3.013 (SE .016) and the slope being −.843 (SE .013) (Table S5). The model fit was good for SRMR only (.049) and gradually improved for the other model fit indexes (except for TLI) as literacy support was added in the analyses (final adjusted model with interaction term for reading support: RMSEA .039, CFI .931, TLI .792, SRMR .015). Table 2 shows the unstandardized associations between literacy difficulties at age 11 and the intercept and slope of leisure reading. In the unadjusted model, we found evidence that literacy difficulties at age 11 were associated with the intercept, but not with the slope, of leisure reading. Specifically, literacy difficulties in childhood were associated with lower initial levels of leisure reading at age 11 (−.504, SE .044, *p* < .001), but not with reading enjoyment over time. When adjusting for key covariates (minimally adjusted model), literacy difficulties at age 11 were still associated with lower initial levels of leisure reading at age 11, but less strongly (−.116, SE .050, *p* < .05).

**TABLE 2:** Models 1A, 2A, 3A—Unadjusted and minimally/fully adjusted models of associations between literacy difficulties at age 11 and trajectories of leisure reading at ages 11, 14 and 17 years (unstandardized results).

	1A unadjusted model	2A minimally adjusted model	3A fully adjusted model
	*b* (SE)	*b* (SE)	*b* (SE)
Leisure reading intercept			
Literacy difficulties age 11	−.504*** (.044)	−.116* (.050)	−.051 (.051)
Leisure reading slope			
Literacy difficulties age 11	.048 (.040)	.039 (.045)	.002 (.046)

Abbreviations: *b*, unstandardized beta coefficient; SE, standard error.

*p < .05; **p < .01; ***p < .001.

### The role of literacy support at age 11 in the relationship between literacy difficulties in childhood and leisure Reading

Next, we sought to examine whether receiving literacy support moderated the relationship between literacy difficulties at age 11 and leisure reading, both concurrently and over time. Chi‐squared tests of association showed that literacy difficulties at age 11 were significantly associated with literacy support at age 11. However, literacy support was not associated with leisure reading at any time point. Analyses were then conducted to examine whether literacy support was a moderator in the association between literacy difficulties at age 11 and later trajectories of leisure reading (Table 3, Tables S6 and S7). We found some evidence that children who received literacy support tended to have lower initial levels of leisure reading compared to those who did not receive support (−.564, SE .265, *p* < .05) in the unadjusted model, but not in the minimally adjusted model. This was not the case for the slope of leisure reading in either model. As for the interaction terms, these were again not significant in either of the models, indicating the presence of literacy support did not moderate the association between literacy difficulties on either the intercept or the slope of leisure reading.

**TABLE 3:** Models 1B, 2B, 3B – Unadjusted and minimally/fully adjusted models of interaction of literacy difficulties at age 11 with literacy support at age 11 in association with trajectories of leisure reading at ages 11, 14 and 17 years (unstandardized results).

	1B unadjusted model	2B minimally adjusted model	3B fully adjusted model
	*b* (SE)	*b* (SE)	*b* (SE)
Leisure reading intercept			
Literacy difficulties age 11	−.509*** (.045)	−.121* (.050)	−.056 (.051)
Literacy support age 11	−.564* (.265)	−.395 (.241)	−.332 (.248)
Literacy difficulties age 11 Literacy support age 11^a^	.630 (.351)	.434 (.315)	.366 (.328)
Leisure reading slope			
Literacy difficulties age 11	.046 (.040)	.038 (.045)	.000 (.046)
Literacy support age 11	.106 (.200)	.123 (.195)	.140 (.193)
Literacy difficulties age 11 Literacy support age 11^a^	.032 (.352)	.010 (.354)	.040 (.360)

Abbreviations: *b*, unstandardized beta coefficient; SE, standard error.

^a^ Interaction term.

*p < .05; **p < .01; ***p < .001.

When checking the variance of the intercept and slope of leisure reading, we found that the standardized residual variance of both parameters was significant in the unadjusted model (intercept: .945, SE .010, *p* < .001; slope: .999, SE .002, *p* < .001), indicating the presence of interindividual differences in how leisure reading developed over time.

### Sensitivity analyses

The analyses checking for the moderating role of literacy support at age 11 were repeated, but this time the association between literacy difficulties at age 11 and trajectories of leisure reading was analysed in a complete cases sample. For the complete case analysis, participants with missing data on literacy difficulties and leisure reading were excluded, resulting in a reduced sample size of *N* = 3464 (Table S8). The only difference from the main analyses was that the association between literacy support at age 11 and the intercept of leisure reading was no longer significant.

We also re‐ran the analyses adjusting for all covariates. In these analyses, literacy difficulties were not significantly associated with the intercept or slope of leisure reading. Similar to the unadjusted and minimally adjusted models, interaction terms were not significant in the fully adjusted model. When checking the variance of the intercept and slope of leisure reading, we found that the standardized residual variance of both parameters was significant in the fully adjusted model (intercept: .749, SE .023, *p* < .001; slope: .977, SE .008, *p* < .001), further indicating the presence of inter‐individual differences in how leisure reading developed over time.

## DISCUSSION

Using data from the MCS, we sought to examine how literacy difficulties in upper primary years (at age 11) were associated with leisure reading concurrently and over time, and whether receiving literacy support moderated this relationship. The findings presented here add to the existing literature in two important ways. First, through examining longitudinal associations between literacy difficulties and leisure reading in older children, and the role of targeted support in this relationship. Second, by looking at the impact of support practices happening in UK schools, rather than within experimental settings.

### Literacy difficulties and leisure Reading over time

In our sample, leisure reading declined over time for all children, with only 13.4% reporting reading for pleasure most days at age 17, compared to almost 50% at age 11. This is in line with existing evidence that as children progress through school, their leisure reading tends to decline (Clark et al., 2024; Miyamoto et al., 2020). Potential explanations have been put forth to explain this trend, including that as children get older, they have less time to read for pleasure compared to an increased focus on reading for performance (e.g., for exams; Miyamoto et al., 2020). Given the many benefits of reading for pleasure, understanding how to counter this decline should be a priority for educational practice.

We also found that literacy difficulties at age 11 were significantly associated with less frequent leisure reading at ages 11, 14 and 17. Torppa et al. (2020) suggested that there is a bi‐directional relationship between reading achievement and leisure reading; in early years, poorer reading comprehension and fluency predict less leisure reading, while in later years, less frequent leisure reading predicts slower growth in reading comprehension. Plausibly, higher initial skills make reading more enjoyable for children, leading them to engage more frequently in leisure reading, while more frequent leisure reading supports growth by providing opportunities to develop key skills such as orthographic knowledge and vocabulary (Torppa et al., 2020). It is therefore possible that literacy difficulties negatively impacted leisure reading at all three ages in our sample.

However, further analyses using growth curve modelling indicated that literacy difficulties at age 11 were associated with less frequent leisure reading at 11 in the unadjusted and minimally adjusted models, but not in the fully adjusted model used in our sensitivity analyses. Namely, when sex, family income, home learning environment, leisure reading at age 7 and reading accuracy were controlled for, literacy difficulties at 11 contributed additional variance in leisure reading at 11. However, when additional covariates of ethnicity, maternal education, number of books at home, cognitive ability and social and behavioural problems were included in the model, literacy difficulties did not predict additional variance in leisure reading. When looking at variables individually, controlling for reading accuracy at age 7 greatly reduced the statistical significance of the relationship between literacy difficulties and leisure reading at 11, while the relationship was no longer significant after controlling for the number of books in the home at age 7. Additionally, when looking at the trajectory of leisure reading over time, our analyses did not find that literacy difficulties at 11 predicted lower reading for pleasure later on, and indicated that within the sample, there was significant variability in how leisure reading developed over time. This variability remained present even after adjusting for all covariates, which could indicate that further factors not considered here impacted the trajectory of leisure reading. Importantly, as latent growth curve models estimate a single trajectory as being representative of the whole sample and do not account for within‐sample variability, it is possible that certain individuals within our sample followed different trajectories.

It has been proposed that multiple risk and resilience factors interact to determine reading ability (Carroll et al., 2025; Catts & Petscher, 2022). Our findings indicate that this may also be the case for leisure reading. This is in line with other findings reporting that various factors contribute to leisure reading; for example, Rogiers et al. (2020) reported that girls, children with higher socioeconomic status and those on academic tracks (i.e., higher performing students) tend to read for pleasure more often. Understanding how to foster leisure reading may thus need a multifaceted approach that considers both cognitive and environmental factors. Future studies could attempt to identify subpopulations to further clarify the different ways in which leisure reading develops over time, notably through growth mixture models that examine trajectories of leisure reading for children with and without a history of literacy difficulties.

### Literacy support and the trajectory of leisure Reading

Further analyses examined whether receiving targeted literacy support at age 11 moderated the relationship between literacy difficulties and the trajectory of leisure reading. It is first worth noting that less than 3% of children with teacher‐reported literacy difficulties at age 11 were reported to receive literacy support. This is both surprising and concerning, given evidence that intervention benefits pupils with literacy difficulties (Vaughn & Wanzek, 2014), guidance in all UK curriculums that pupils experiencing difficulties should be supported through targeted literacy support where necessary, and findings that a large proportion of pupils with literacy difficulties fail to meet expected standards in reading and writing (DfE, 2022). Given the small proportion of pupils who received literacy support, and the relatively broad nature of the item used to assess literacy support, findings from these analyses should be treated with caution and are best understood as preliminary. Further research is needed to more robustly examine how different school‐based literacy support practices moderate the relationship between literacy difficulties and leisure reading.

Our findings did not provide evidence that literacy support received in upper primary school impacted leisure reading at the time of receiving support or that, over time, children who received literacy support had a different trajectory of leisure reading than those who did not receive support. While it is possible that receiving support positively impacted achievement, which was not measured here, this indicates that receiving support did not have an impact on leisure reading for the children in our sample. Our findings are in line with those of Quirk and Schwanenflugel (2004), who reported that existing remedial reading interventions do not improve reading motivation. They argue that this is caused by a failure to explicitly target motivational mechanisms within interventions, such as goal setting, retraining performance attributions or supporting intrinsic interest. While the MCS doesn't provide detailed information on the support approaches used, based on curriculum guidance during the time period examined (i.e., 2011 onwards), it is likely that the type of support delivered in UK schools focused primarily on developing reading skills (e.g., through SSP approaches; Solity, 2022). Findings presented here suggest that existing targeted support approaches, overall, are not in and of themselves sufficient to promote a long‐term habit of leisure reading.

If supported by future research, these preliminary findings indicate a shift may be needed in how we support pupils with persistent literacy difficulties, with motivation incorporated as an explicit focus of instruction. There is evidence that such an approach would benefit pupils. Meta‐analyses of reading interventions that focus explicitly on supporting motivation have found that these have a significant impact on both motivational and performance outcomes (e.g., Guthrie et al., 2007; McBreen & Savage, 2021; van der Sande et al., 2023). Further, meta‐analyses examining interventions for struggling readers specifically have found that compared to interventions that target only reading skills, those that target both skills and motivation lead to greater progress in reading for children with literacy difficulties across primary school (e.g., Cho et al., 2023; McBreen & Savage, 2022). Notably, interventions that include explicit instruction in motivational strategies (e.g., goal setting, progress monitoring) tend to yield greater benefits compared to those which only include motivational supports (e.g., providing choice, game‐based learning) (Cho et al., 2023).

### Limitations

The present study is unique in using cohort data to examine the role of school‐based support and student reports of leisure reading at later timepoints. However, when speculating about the nature of these relationships and implications of the findings, several limitations should be considered. First, despite drawing upon a large initial sample of pupils with teacher‐reported literacy difficulties at age 11 (*n* = 1463), only 2.3% of this sample (*n* = 33) received literacy support. It is possible that receiving support may have an impact on enjoyment, but that the sample was underpowered to detect effects. Second, there was a large proportion of missing data (close to 50%) for leisure reading at age 17, which may have similarly limited the possibility of our analyses to detect effects. This was mitigated through the use of a FMIL approach, allowing the analysis to be completed on the full analytic sample for all models. Third, due to the nature of MCS data, it was not possible to gain insight into the specific content of literacy support that pupils received. Nonetheless, the findings reported here suggest that simply receiving support did not in and of itself foster greater leisure reading at later time points. Future research should investigate the specific components of reading support interventions to identify the elements that are more or less effective in improving leisure reading in children with literacy difficulties. Fourth, we used a teacher‐reported measure to identify children with literacy difficulties to be included in the sample. It is possible that use of a subjective measure led to inclusion of pupils without literacy difficulties or exclusion of some pupils with literacy difficulties from the sample. However, it is likely to have led to inclusion of all pupils who would have been deemed eligible to receive literacy support and was deemed more appropriate than a diagnosis‐based inclusion criterion, due to differential access to dyslexia diagnosis in the United Kingdom (Knight & Crick, 2021). Finally, the measures of leisure reading were collected using five‐ or six‐point Likert scales, which may have led to ceiling or floor effects that limited the ability to detect changes over time with sufficient sensitivity.

### Conclusions and directions for future research

While curriculums in the UK mandate that all schools should seek to foster a love of reading for pupils, how effectively this is done in practice remains unknown (Cremin & Scholes, 2024) and persistent declines in leisure reading indicate more research in this area is urgently needed (Clark et al., 2024). The findings presented here have been a useful first step towards exploring the impact of school practices on leisure reading. They suggest that a steep decline in reading for pleasure occurs for pupils with literacy difficulties as they progress through the end of primary and secondary school, irrespective of whether they receive targeted literacy support in upper primary years or not. The factors contributing to this decline are likely to be complex and to result from interactions between individual and environmental factors, in line with previous work acknowledging multiple individual and contextual factors impacting leisure reading (Cremin & Scholes, 2024). Future research that examines the nature of school‐based literacy support and actively tracks its impact on leisure reading longitudinally is needed to fully understand the impact of different approaches, while research that combines analysis of overall trends with within‐person analyses is needed to better understand for whom and under which conditions targeted literacy support is most effective (e.g., Konen & Karbach, 2015). More broadly, future research that examines how educators and parents can foster leisure reading, and that considers how this can be incorporated within school settings effectively, is needed.

## AUTHOR CONTRIBUTIONS

**Miriam McBreen:** Conceptualization; investigation; writing – original draft; methodology; writing – review and editing; project administration; supervision. **Francesca Bentivegna:** Writing – original draft; methodology; writing – review and editing; formal analysis; data curation; investigation. **Emma Sumner:** Investigation; writing – original draft; writing – review and editing. **Efstathios Papachristou:** Writing – review and editing; writing – original draft; methodology; formal analysis; supervision.

## CONFLICT OF INTEREST STATEMENT

The authors have no conflicts of interest to declare for this article.

## Supporting information


Tables S1–S8.

## Data Availability

The data that support the findings of this study are openly available in UK Data Service at https://datacatalogue.ukdataservice.ac.uk/series/series/2000031#abstract, reference number doi: http://doi.org/10.5255/UKDA‐Series‐2000031.
