# Supplementary material for: Trajectories of leisure reading for children with literacy difficulties and the role of literacy support: Findings from the UK millennium cohort study
Source: Br J Educ Psychol. 2026 Aug 5;96(4):1769–84. doi: 10.1111/bjep.70113 (PMC13752610; doi:10.1111/bjep.70113)
Supplement: Supplementary file 1 [file BJEP-96-1769-s001.docx]

**Supplementary tables.**

**Table S1.**

*Fit indices for latent growth models in the analytic sample*

|  | RMSEA | CFI | TLI | SRMR |
| --- | --- | --- | --- | --- |
|  |  |  |  |  |
| Trajectory-only latent growth model | 0.151 | 0.869 | 0.607 | 0.049 |
| Unadjusted model with literacy support | 0.067 | 0.904 | 0.711 | 0.030 |
| Minimally adjusted model with literacy support | 0.046 | 0.925 | 0.774 | 0.019 |
| Fully adjusted model with literacy support | 0.039 | 0.931 | 0.792 | 0.015 |

**Table S2.**

*Fit indices for the complete case sensitivity analysis*

|  | RMSEA | CFI | TLI | SRMR |
| --- | --- | --- | --- | --- |
|  |  |  |  |  |
| Trajectory-only latent growth model | 0.133 | 0.940 | 0.821 | 0.035 |
| Unadjusted model with literacy support | 0.060 | 0.951 | 0.852 | 0.024 |
| Minimally adjusted model with literacy support | 0.044 | 0.951 | 0.852 | 0.017 |
| Fully adjusted model with literacy support | 0.038 | 0.954 | 0.861 | 0.014 |

**Table S3.**

*Cross-tabulation*

|  | **Literacy support (age 11)** | | **Leisure reading (Age 11)** | | | | | **Leisure reading (Age 14)** | | | | | **Leisure reading (Age 17)** | | | | |
| --- | --- | --- | --- | --- | --- | --- | --- | --- | --- | --- | --- | --- | --- | --- | --- | --- | --- |
|  | No | Yes | Never or almost | Less than once a month | At least once a month | At least once a week | Most days | Never or almost | Less than once a month | At least once a month | At least once a week | Most days | Never or almost | Less than once a month | At least once a month | At least once a week | Most days |
| **Literacy difficulties (Age 11)** |  |  |  |  |  |  |  |  |  |  |  |  |  |  |  |  |  |
| No | 5,559 | 32 | 282 | 380 | 563 | 1,657 | 2,836 | 777 | 1,170 | 901 | 911 | 1,163 | 816 | 1,051 | 491 | 402 | 439 |
| Yes | 1,388 | 33 | 198 | 132 | 152 | 430 | 522 | 338 | 307 | 162 | 165 | 181 | 254 | 143 | 59 | 65 | 65 |
| **Literacy support (Age 11)** |  |  |  |  |  |  |  |  |  |  |  |  |  |  |  |  |  |
| No | / | / | 457 | 491 | 692 | 2,019 | 3,238 | 1,065 | 1,433 | 1,031 | 1,045 | 1,296 | 1,033 | 1,152 | 526 | 447 | 492 |
| Yes | / | / | 7 | 8 | 8 | 15 | 27 | 15 | 12 | 7 | 8 | 10 | 8 | 7 | 7 | 4 | 2 |

**Table S4.**

*Unweighted sample bias analysis*

| **Exposure and outcomes** | **Analytic sample**  **(N = 7,291)** | | **Non-analytic sample**  **(N = 11,952)** | | **Test of difference** |
| --- | --- | --- | --- | --- | --- |
|  | **N** | **%** | **N** | **%** | **X^2^** |
| **Literacy difficulties (Age 11)** |  |  |  |  | 27.64*** |
| No literacy difficulties | 5,770 | 79.8 | 21 | 47.7 |  |
| Literacy difficulties | 1,463 | 20.2 | 23 | 52.3 |  |
| **Literacy support support (Age 11)** |  |  |  |  | 0.41 |
| No reading support | 6,974 | 99.1 | 44 | 100.0 |  |
| Reading support | 65 | 0.9 | 0 | 0.0 |  |
| **Leisure reading (Age 11)** |  |  |  |  | 31.65*** |
| Never of almost never | 483 | 6.7 | 478 | 8.4 |  |
| Less often than once a month | 518 | 7.2 | 447 | 7.8 |  |
| At least once a month | 719 | 10.0 | 662 | 11.6 |  |
| At least once a week | 2,109 | 29.2 | 1,652 | 29.0 |  |
| Most days | 3,381 | 46.9 | 2,463 | 43.2 |  |
| **Leisure reading (Age 14)** |  |  |  |  | 34.70*** |
| Never of almost never | 1,122 | 18.3 | 1,171 | 22.5 |  |
| Less often than once a month | 1,486 | 24.3 | 1,279 | 24.6 |  |
| At least once a month | 1,073 | 17.5 | 844 | 16.2 |  |
| At least once a week | 1,092 | 17.8 | 868 | 16.7 |  |
| Most days | 1,351 | 22.1 | 1,044 | 20.0 |  |
| **Leisure reading (Age 17)** |  |  |  |  | 17.48** |
| Never of almost never | 1,078 | 28.3 | 956 | 32.8 |  |
| Less often than once a month | 1,203 | 31.5 | 883 | 30.3 |  |
| At least once a month | 553 | 14.5 | 389 | 13.3 |  |
| At least once a week | 472 | 12.4 | 315 | 10.8 |  |
| Most days | 510 | 13.4 | 374 | 12.8 |  |
| **Covariates (binary/categorical)** |  |  |  |  |  |
| **Child’s sex** |  |  |  |  | 9.26** |
| Male | 3,647 | 50.0 | 6,242 | 52.3 |  |
| Female | 3,644 | 50.0 | 5,698 | 47.7 |  |
| **Child’s ethnicity** |  |  |  |  | 0.094 |
| White | 5,969 | 81.9 | 9,763 | 81.7 |  |
| Other than white | 1,322 | 18.1 | 2,188 | 18.3 |  |
| **Family income^c^** |  |  |  |  | 87.30*** |
| Above 60% median | 4,944 | 73.7 | 4,725 | 66.4 |  |
| Below 60% median | 1,766 | 26.3 | 2,392 | 33.6 |  |
| **Maternal education^c^** |  |  |  |  | 53.45*** |
| No higher education | 4,117 | 61.3 | 4,792 | 67.3 |  |
| Higher education | 2,599 | 38.7 | 2,333 | 32.7 |  |
| **Number of books at home^d^** |  |  |  |  | 66.64*** |
| 0-10 | 935 | 13.0 | 945 | 16.2 |  |
| 11-25 | 970 | 13.5 | 939 | 16.1 |  |
| 26-100 | 2,358 | 32.8 | 1,893 | 32.5 |  |
| 101-200 | 1,472 | 20.4 | 1,017 | 17.5 |  |
| 201-500 | 1,023 | 14.2 | 742 | 12.7 |  |
| More than 500 | 443 | 6.1 | 291 | 5.0 |  |
| **Reading enjoyment^c^** |  |  |  |  | 5.32 |
| Don’t like it | 682 | 10.9 | 791 | 12.1 |  |
| Like it a bit | 1,943 | 31.1 | 2,056 | 31.4 |  |
| Like it a lot | 3,626 | 58.0 | 3,694 | 56.5 |  |
| **Covariates (continuous)** | N | M (SD) | N | M (SD) | F |
| Home learning environment^a^ | 6,144 | 26.92 (6.90) | 7,807 | 26.20 (7.00) | 37.57*** |
| Child’s cognitive ability^#b^ | 6,758 | 101.30 (14.51) | 8,105 | 98.92 (15.31) | 92.96*** |
| Reading accuracy^#c^ | 6,552 | 101.72 (14.69) | 6,850 | 98.35 (15.10) | 171.41*** |
| Mental health difficulties total score^c^ | 6,615 | 6.98 (4.98) | 6,923 | 7.38 (5.24) | 19.92** |

^a^ measured at age 3; ^b^ measured at age 5; ^c^ measured at age 7; ^d^ measured at age 11. ^#^standardised.

*Note.* * p < .05; ** p < .01; *** p < .001

N = Total number of cohort members; *%* = Percentages with number of non-missing values as the denominator; M = Mean; SD = Standard Deviation; X^2^ = Pearson’s Chi-Square; F = One-way ANOVA.

**Table S5.**

*Leisure reading trajectories*

|  | **Estimate** | **SE** | **Two-Tailed P-Value** |
| --- | --- | --- | --- |
| **Model Means** |  |  |  |
| Leisure reading Intercept | 3.013 | 0.016 | 0.000 |
| Leisure reading Slope | -0.843 | 0.013 | 0.000 |
| **Growth parameters correlation (standardised)** |  |  |  |
| Leisure reading Intercept |  |  |  |
| Leisure reading Slope | -0.296 | 0.046 | 0.000 |

SE = Standard error

**Table S6.**

*Unadjusted and minimally/fully adjusted models of associations between literacy difficulties at age 11 and trajectories of leisure reading at ages 11, 14, and 17 years (standardised results).*

|  | **Unadjusted model** | **Minimally adjusted** | **Adjusted model** |
| --- | --- | --- | --- |
|  | **b (SE)** | **b (SE)** | **b (SE)** |
| ***Leisure reading intercept*** |  |  |  |
| Literacy difficulties age 11 | -0.229*** (0.021) | -0.054* (0.023) | -0.024 (0.024) |
| ***Leisure reading slope*** |  |  |  |
| Literacy difficulties age 11 | 0.032 (0.026) | 0.027 (0.030) | 0.001 (0.031) |

*Note.* ****p* < .05; ** *p* < .01; *** *p* < .001.**

*b* = Unstandardised beta coefficient; *SE* = standard error.

**Table S7.**

*Unadjusted and minimally/fully adjusted models of interaction of literacy difficulties at age 11 with literacy support at age 11 in association with trajectories of leisure reading at ages 11, 14, and 17 years (standardised results).*

|  | **Unadjusted model** | **Minimally adjusted model** | **Fully adjusted model** |
| --- | --- | --- | --- |
|  | **b (SE)** | **b (SE)** | **b (SE)** |
| ***Leisure reading intercept*** |  |  |  |
| Literacy difficulties age 11 | -0.231*** (0.021) | -0.056* (0.023) | -0.026 (0.024) |
| Literacy support age 11 | -0.062 (0.029) | -0.044 (0.027) | -0.037 (0.028) |
| Literacy difficulties age 11  # Literacy support age 11 | 0.046 (0.026) | 0.032 (0.023) | 0.027 (0.024) |
| ***Leisure reading slope*** |  |  |  |
| Literacy difficulties age 11 | 0.031 (0.027) | 0.026 (0.031) | 0.000 (0.031) |
| Literacy support age 11 | 0.017 (0.032) | 0.020 (0.032) | 0.023 (0.032) |
| Literacy difficulties age 11  # Literacy support age 11 | 0.003 (0.038) | 0.001 (0.039) | 0.004 (0.039) |

*Note.* **p* < .05; ** *p* < .01; *** *p* < .001.

b = beta coefficient; *SE* = Standard error; # = Interaction term.

**Table S8.**

*Complete case analysis (N=3,464) – Unadjusted and minimally/fully adjusted models of interaction of literacy difficulties at age 11 with literacy support at age 11 in association with trajectories of leisure reading at ages 11, 14, and 17 years (unstandardised results).*

|  | **Unadjusted model** | **Minimally adjusted model** | **Fully adjusted model** |
| --- | --- | --- | --- |
|  | **b (SE)** | **b (SE)** | **b (SE)** |
| ***Leisure reading intercept*** |  |  |  |
| Literacy difficulties age 11 | -0.441*** (0.067) | -0.102 (0.073) | -0.052 (0.075) |
| Literacy support age 11 | -0.401 (0.323) | -0.284 (0.324) | -0.166 (0.329) |
| Literacy difficulties age 11  # Literacy support age 11 | 0.497 (0.493) | 0.409 (0.473) | 0.337 (0.489) |
| ***Leisure reading slope*** |  |  |  |
| Literacy difficulties age 11 | 0.017 (0.048) | -0.001 (0.053) | -0.033 (0.054) |
| Literacy support age 11 | 0.033 (0.232) | 0.045 (0.230) | 0.062 (0.227) |
| Literacy difficulties age 11  # Literacy support age 11 | 0.265 (0.433) | 0.248 (0.437) | 0.265 (0.441) |

*Note.* **p* < .05; ** *p* < .01; *** *p* < .001.

*b* = Unstandardised beta coefficient; *SE* = Standard error; # = Interaction term.
